# Ethnobotanical Research and Compilation of the Medicinal Uses in Spain and the Active Principles of *Chiliadenus glutinosus* (L.) Fourr. for the Scientific Validation of Its Therapeutic Properties

**DOI:** 10.3390/plants10030584

**Published:** 2021-03-19

**Authors:** Nadia Las Heras Etayo, Félix Llamas, Carmen Acedo

**Affiliations:** Biodiversity and Environment Management Department, Faculty of Biological and Environmental Sciences, University of León, 24007 León, Spain; f.llamas@unileon.es

**Keywords:** *Chiliadenus glutinosus*, ethnopharmacological, bioactive phytochemicals, in silico modeling, human body organic systems, Spanish folk medicine

## Abstract

The species *Chiliadenus glutinosus* (L.) Fourr. has a large number of therapeutic uses reported in the traditional Spanish medicine. The growing interest in preserving the ethnopharmacological knowledge related to the botanical diversity existing in Spain and the interest in achieving scientific validation of the therapeutic properties of medicinal species has led to the development of this study. To do it, all the known medicinal uses of *Ch. glutinosus* in Spain were compiled, then an exhaustive bibliographic research on its chemical composition was carried out, and finally, an in silico validation of the bioactive phytochemicals present in a higher proportion in the essential oil of *Ch. glutinosus*: camphor, borneol, lucinone, glutinone, quercetin, kutdtriol, and kaempferol; in an attempt to justify the reported traditional uses of the species. It was found that much of the traditional medicinal uses of *Ch. glutinosus*, along with the biological activity of its phytochemicals, are supported by scientific evidence. The results place this species in a prominent position to initiate possible lines of research to develop new, more effective drugs and improve therapies to treat conditions and diseases that affect the different organic systems of the human being.

## 1. Introduction

The genus *Chiliadenus* Cass. belongs to the Asteraceae family. This genus is composed of at least ten endemic species of certain areas of the Mediterranean region. In Spain lives a single species of this genus, *Chiliadenus glutinosus* (L.) Fourr., whose distribution in the Eastern Spanish mainland also extends through the island of Mallorca, southern France, and northern Morocco [1,2,3,4,5,6]. 

Medicinal plants have been used by mankind since ancient times as a remedy for all kinds of ailments and conditions, especially human remedies. However, *Chiliadenus glutinosus*, popularly known as “té de roca” (rock tea), “té de Aragon” (Aragon tea), or “arnica” [7], has not been referenced for its medicinal uses in past centuries by botanists such as Laguna [8], Clusius [9,10], or Quer y Martínez [11]. Palau y Verdera [12] cites it by a synonym in the 18th century, without mentioning any traditional use; and at the end of the 19th century, the Spanish botanists Loscos Bernal and Pardo Sastrón [13,14], and also the German, Gadow [15], collected the first references on the therapeutic uses of the species. This plant is also not listed in the Royal Spanish Pharmacopoeia [16]. This contrasts with its known and widespread use today and in recent decades in those places in Spain where the species grows naturally, mainly in some regions of the eastern half of the peninsula [1,17]. 

*Chiliadenus glutinosus* is a plant with a wide range of medicinal applications in Spanish popular culture. Remedies based on this species have been used to treat problems of the circulatory system (to regulate tension, purify the blood, improve the heart, etc.), digestive problems (to relieve stomach pains, ulcers, indigestion, etc.), genito-urinary disorders (to remove kidney stones or to help urination), to treat problems of the respiratory system (colds, sore throats, asthma, etc.), improve conditions of the locomotor system (rheumatic pains, or blows), also of the nervous system (headaches, as a tranquilizer, etc.), and finally, treat conditions of the integumentary system (disinfection of wounds, burns, etc.) [16,17,18,19,20,21,22,23]. 

Several studies on the chemical composition of *Chiliadenus glutinosus* [24,25,26] have been previously published, confirming the presence of a wide variety of phytochemicals, of which about one hundred different compounds have been described to date. In this study, the medicinal uses of *Ch. glutinosus* in Spain will be compiled and its chemical composition will be reviewed to validate or refute the therapeutic properties attributed to the species in Spanish folk medicine, which will be carried out using a bibliographic review of clinical trials and in silico simulation to predict the molecular targets of the phytochemicals present in the plant.

## 2. Results

### 2.1. Description of Chiliadenus glutinosus (L.) Fourr.

**Figure 1:**
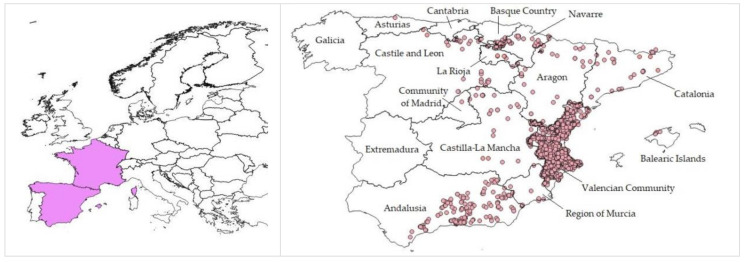
Distribution map of the species *Chiliadenus glutinosus* (L.) Fourr. (left) and detail in Spain produced in the ArcGis software from data obtained in GBIF [27,28], corrected according the last revision of the Flora Iberica [7].

*Chiliadenus glutinosus* is a species of the Asteraceae family, distributed throughout the eastern half of the Iberian Peninsula (Figure 1) and used popularly for various therapeutic purposes. Its presence is confirmed in 34 of the 50 provinces, and 14 of the 17 regions (Autonomous Communities), that make up the national territory, and medicinal uses of the species have been reported in at least 20 provinces [7,17]. 

**Figure 2:**
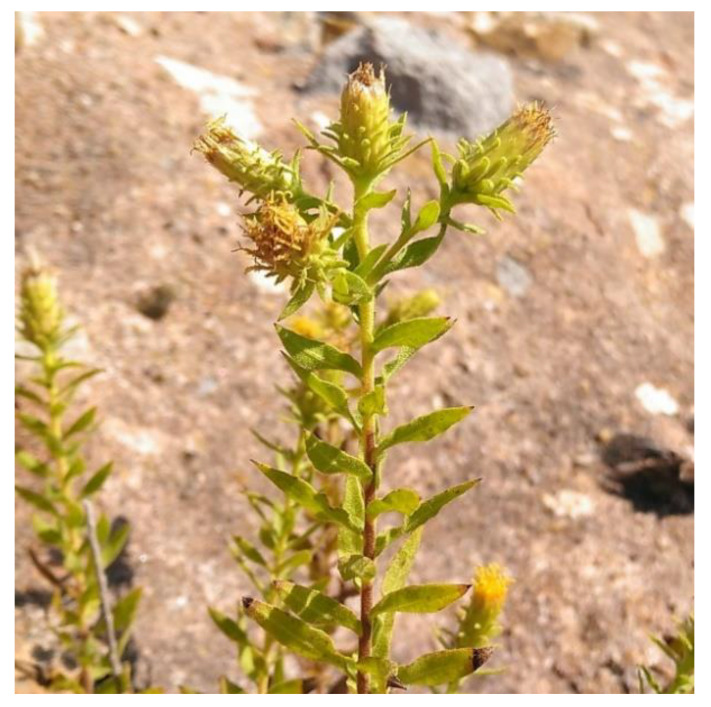
Flowering top of *Chiliadenus glutinosus* used in folk medicine and detail of the mature inflorescences. Photograph was taken in the town of Arnedillo (La Rioja).

It is a perennial plant with a size between 10 and 45 cm with a simple or branched stem, and abundant trichomes, some short glandular (up to 0.5 mm), and others long non-secreting (0.2–5 mm). It has a narrow rhizome with long and thin roots that are introduced between the rocks. The leaves are 10–25 × 2–5 mm in size and are lanceolate, sharp, erect-patent and, covered with glandular hairs. The flower heads are 7–15 mm wide and are gathered in corymbose or cymose inflorescences, sometimes also solitary. The involucre is 8–15 mm long and has bracts in several rows. The external bracts (3–5 × 0.5–1 mm) are herbaceous, secretory, and considerably shorter than the internal ones. The internal bracts (5–10 × 0.7–1 mm) are scarious, ciliated, and non-glandular. In each flower head, there are between 55 and 105 yellow florets, and there are no ray florets. The cypsela is pubescent with glandular hairs at the apex. The pappus has two rows of hairs, the outer one with short hairs (0.5–1 mm) and the inner one with longer hairs (5–7.5 mm), denticulate, and reddish-brown (Figure 2). It is a basophilic species, lives exclusively in fissures and cracks of sunny calcareous crags at between 350 and 1000 meters of altitude, and its flowering takes place between July and August [7,29,30,31].

### 2.2. Compilation of the Medicinal Uses of Chiliadenus glutinosus

The medicinal uses of the species *Chiliadenus glutinosus* in Spanish culture have been compiled by reviewing specific literature and are set out in the following Tables 1–7, arranged into Regions (the Spanish Autonomous Community). The part used to prepare these remedies is usually the flowering top of the plant, sometimes the part used is not specified.

#### 2.2.1. Medicinal Uses Applied to the Human Circulatory System

Ten different medicinal uses of *Chiliadenus glutinosus* have been compiled aimed at treating different disorders of the human circulatory system in six Autonomous Communities. Its use is mentioned in the form of an infusion to regulate blood pressure, in Catalonia with a hypertensive effect and in north of Andalusia with a hypotensive effect. In Aragon, southeast of Castilla-La Mancha and north of Andalusia, the infusion is taken to give fluidity to the blood and improve its circulation. This infusion is also used to treat other related problems, such as purifying the blood in Navarra, relieving pain in swollen legs, or improving circulation in north of Valencian Community and Region of Murcia, and fighting anemia in Region of Murcia. Finally, it is recommended to take this infusion to people with weak hearts or varicose vein problems in southeast of Castilla-La Mancha, and for heart ailments in general in north of Andalusia (Table 1).

#### 2.2.2. Medicinal Uses Applied to the Human Digestive System

A total of eleven medicinal uses of *Chiliadenus glutinosus* have been collected from thirteen Spanish Autonomous Communities, aimed at treating conditions of the digestive system. In some areas of Navarre and Catalonia, the plant is left to macerate in anise for some time and the resulting liquid is drunk as a digestive drink after meals. Another of the most popular uses of the plant is its use as a digestive infusion in a wide territory comprised of several regions: east of Catalonia, Aragon, Navarre, southern Basque Country, La Rioja, southeast of Castile and Leon, southeast of Castilla-La Mancha, center and south of Valencian Community, Cantabria, and towns in the northeast and southeast of Andalusia. *Ch. glutinosus* infusion is used as a remedy for diarrhea in north of Aragon, Navarre, southern Basque Country, north and center of Valencian Community, and north of Andalusia; for appendicitis in north of Aragon, and to relieve stomach pain in Navarre, southern Basque Country), center of Valencian Community, Community of Madrid, and southeast of Castilla-La Mancha. In other towns of belonging to southeast of Castilla-La Mancha, Region of Murcia, and north of Andalusia it is recommended to take this infusion for several days to cure stomach ulcers; in center of Valencian Community and north of Andalusia it is also used to eliminate gasses, and in Region of Murcia, it is recommended to reduce the feeling of heaviness in the stomach. This infusion is also used to stimulate the appetite in center of Valencian Community and Region of Murcia or to induce vomiting in high doses in north of Valencian Community. In other regions of Spain such as south of Aragon, east of Castilla-La Mancha, center of Valencian Community, northeast and southeast of Andalusia or the Balearic Islands, the infusion is recommended in a more non-specific way for all types of stomach problems and indispositions (Table 2).

#### 2.2.3. Medicinal Uses Applied to the Human Genitourinary System

The five medicinal uses of *Chiliadenus glutinosus* compiled to treat the genitourinary system are prepared in infusion, in the four different Autonomous Communities. The infusion of this plant is taken to help urinate more frequently, since it is considered to be diuretic, in towns in Region of Murcia, southeast of Castilla-La Mancha, and Cantabria. Moreover, in Region of Murcia and southeast of Castilla-La Mancha, it is recommended to take this infusion when suffering from kidney pains or to promote the elimination of kidney stones. Finally, in some towns of Region of Murcia and north of Andalusia, it is recommended to drink the infusion to treat kidney diseases in general and to “improve the kidney” by taking the infusion preventively to avoid possible infections, kidney stones, etc. (Table 3).

#### 2.2.4. Medicinal Uses Applied to the Human Locomotor System

Three medicinal uses of *Chiliadenus glutinosus* have been compiled in three Autonomous Communities. The plant is applied as a decoction, macerated in alcohol, and as an infusion to treat conditions of the locomotor system, mainly to combat rheumatic and bone pain. In locations of north of Andalusia, the plant is cooked, and the resulting liquid is applied in the form of baths in the joint areas where pain is suffered, and in the Region of Murcia, the fresh plant is collected and left to macerate in alcohol for some time. Then, that alcohol is applied in the form of rubbing to relieve sore areas from rheumatism. Finally, the plant is also used as an internal infusion to relieve body fatigue in southeast of Castilla-La Mancha (Table 4).

#### 2.2.5. Medicinal Uses Applied to the Human Nervous System

A total of four medicinal uses of *Chiliadenus glutinosus* have been compiled in infusion form, in five Autonomous Communities, aimed at treating conditions of the human nervous system. In some localities of northwest and northeast of Catalonia, center of Valencian Community and southeast of Castilla-La Mancha, the plant is prepared in infusion and it is taken to calm the nerves. In Navarre, other properties are attributed to it and this infusion is considered to lift mood and clear the mind; also, in Navarre and some municipalities of east of Castilla- La Mancha and north of Andalusia, it is recommended to drink this infusion to relieve headaches (Table 5).

#### 2.2.6. Medicinal Uses Applied to the Human Respiratory System

For the respiratory system, six medicinal uses of *Chiliadenus glutinosus* have been compiled, using the plant in an infusion, decoction, or vapors, in eight Autonomous Communities, as a remedy for diseases of the human respiratory system. The use of the plant in infusion has been reported in numerous towns in northwest and east of Catalonia, Aragon, Navarre, center of Valencian Community, Region of Murcia, southeast of Castilla-La Mancha, north of Andalusia, and Cantabria, as a good remedy for colds and flu. Other applications of this infusion are to relieve a sore throat in northwest of Catalonia, Region of Murcia, and north of Andalusia; treat bronchitis in southeast of Castilla-La Mancha and north of Andalusia; or improve asthmatic processes in southeast of Castilla-La Mancha. Another of the reported uses of this plant has been the use of the vapors resulting from its decoction as a remedy to open the lungs in cases of asthma in localities of southeast of Castilla-La Mancha. In the Balearic Islands, the decoction or infusion of the plant is also taken to treat respiratory infections in general (Table 6).

#### 2.2.7. Medicinal Uses Applied to the Human Integumentary System

A total of five medicinal uses of *Chiliadenus glutinosus* have been compiled, applied in the form of poultices, washes, ointments, or plasters. These uses have been reported in eight Autonomous Communities and have been aimed at treating conditions of the integumentary system. The fresh leaves of the plant have been applied in a poultice as a remedy for skin pimples in Navarre. Other reported uses are the use of the species as an infusion or decoction to wash and disinfect wounds and bruises in Region of Murcia and southeast of Castilla-La Mancha; the preparation of a healing ointment with the plant to apply also on wounds and bumps on the skin, and in the form of a decoction to relieve the discomfort caused by the insect bites such as mosquitoes in Albacete. In other locations in southeast and east of Castilla-La Mancha, plasters are prepared to treat skin burns or the resulting decoction is applied to them; also, in north of Andalusia, the species is used in the form of decoction or infusion to reduce inflammation of skin wounds (Table 7).

### 2.3. Validation of the Pharmacological Effects of Chiliadenus glutinosus

#### 2.3.1. Phytochemicals from *Chiliadenus glutinosus*

**Figure 3:**
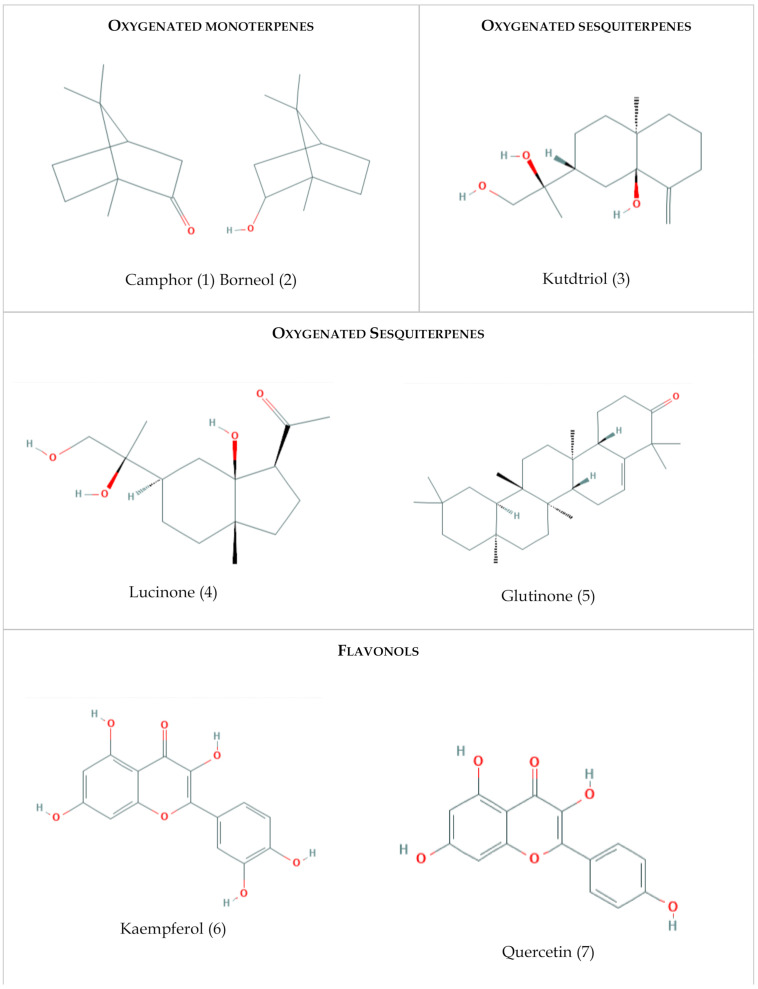
Chemical structure in a two-dimensional format of the main compounds present in *Chiliadenus glutinosus*. Images obtained from NCBI [62,63,64,65,66,67,68].

Several investigations on the chemical composition of *Chiliadenus glutinosus* have been published previously and have identified about one hundred different chemical compounds [24,25,26]. In the present study, it has been decided to select and analyze the following seven phytochemicals: camphor, borneol, lucinone, glutinone, kutdtriol, quercetin, and kaempferol, from the total of compounds, as they are present in greater proportions in the essential oil of *Ch. glutinosus*. The study of the biological activity of the selected phytochemicals could contribute to support or refute the traditional medicinal uses of the species in Spain. Hereunder, Figure 3 below shows the selected compounds, the chemical class to which they belong, and their structure in a two-dimensional format.

#### 2.3.2. Experimental Studies on the Biological Activity of Phytochemicals Present in *Chiliadenus glutinosus*

The Table 8 shows the seven selected compounds present in greater proportions in the essential oil of *Chiliadenus glutinosus* and their biological activities, which could explain the reported medicinal uses of the plant in Spain. Among the compounds listed in Table 8, the anti-inflammatory, antioxidant, and antibacterial activities stand out, proven in clinical trials, of the camphor and borneol monoterpenes, and the quercetin and kaempferol flavonols.

#### 2.3.3. In Silico Modeling for the Prediction of Bioactive Compound Targets

The Swiss Target Prediction software [102] was used to estimate the macromolecular targets most likely to bind to the bioactive compounds camphor, borneol, lucinone, glutinone, kutdtriol, quercetin, and kaempferol, present in *Chiliadenus glutinosus.* This tool statistically quantifies the probability that two similar bioactive molecules share their protein targets. Therefore, when performing a query on the possible binding targets of a specific bioactive molecule, the tool displays and arranges the possible molecular targets from highest to lowest binding probability, up to a maximum of one hundred targets. At times, there can be a 100% binding probability. In these cases, the similarity is total. Therefore, it is likely that the experimental bioactivity of the consulted molecule is known, and it is not a real prediction. What can also happen is that no objective is obtained in the prediction. On this occasion, the input molecule is below the thresholds of similarity in 2D and 3D with any of the known active molecules against which were examined and compared. 

##### Camphor

The camphor molecule was introduced into the Swiss Target Prediction [102] simulator, which assumes it is a bioactive molecule and calculates the binding targets. As a result, 22 camphor-binding targets were obtained in *Homo sapiens*, with binding probabilities between 4% and 20%. The first six results shown in Table 9 have a probability of between 10% and 20% of being targets of the camphor molecule.

Of all the targets obtained, the nuclear receptor of subfamily 1, group I, member 3 (20%), cytochrome P450 (19%), carbonic anhydrases I, II, and IV (15%), and an androgen receptor (10%) showed the highest probability of being targets of the camphor molecule. Data for targets with binding probabilities of less than 10% are not shown.

##### Borneol

Twenty-eight binding targets were obtained in the case of the borneol molecule, in *Homo sapiens*, with binding probabilities between 4% and 35%. The first 12 results obtained have a probability of between 10% and 35% of binding to the borneol molecule. In this case, only the first four results with the highest probability of binding to the borneol are shown in Table 10, which shows probabilities between 31% and 35%. The rest of the targets with binding probabilities lower than 30% are not shown. The first results show as main borneol, the carbonic anhydrases I, II, and IV (36%), and a transient receptor potential cation channel, subfamily M, member 8 (31%).

##### Glutinone

In the case of the glutinone molecule, 32 binding targets were obtained in *Homo sapiens*, with binding probabilities between 12% and 100%. The first three results shown in Table 11 have been selected since they have a probability of between 23% and 100% of being targets of the glutinone molecule. The remaining results for targets with binding probabilities less than 23% are not displayed. Of all the targets analyzed, cycloxigenase 1 (100%), cytochrome P450 (54%), and cathepsin D (23%), showed the highest probability of being glutinone binding targets.

##### Quercetin

For the quercetin molecule, 100 binding targets were obtained in *Homo sapiens*, with binding probabilities between 21% and 100%. The first 67 results have a 100% join probability. In Table 12, the first six targets, NADPH oxidase 4, the vasopressin receptor V2, aldose reductase, xanthine dehydrogenase, monoamine oxidase A, and the insulin-like growth factor I receptor, all with a 100% binding probability. The rest of the objectives are not shown. 

##### Kaempferol

In the case of the kaempferol molecule, 100 binding targets were obtained in *Homo sapiens*, with binding probabilities between 17% and 100%. The first 17 results have a binding probability of 100%. In Table 13, the first six targets have been selected as examples: NADPH oxidase 4, aldose reductase, xanthine dehydrogenase, tyrosinase, tyrosine-protein kinase receptor FLT3, and carbonic anhydrase II. All these targets showed a 100% binding probability with the kampferol, the rest of the results are not shown. Finally, it is worth mentioning that the simulations with the lucinone and kutdtriol molecules did not give results, so it has not been possible to predict the binding targets of these compounds in *Homo sapiens.*

## 3. Discussion

*Chiliadenus glutinosus* is a well-known and commonly used plant for medicinal purposes for decades until the present day throughout eastern Spain. Numerous uses have been reported to treat different pathologies and conditions of the different systems of the human body: the circulatory, digestive, genito-urinary, locomotive, nervous, respiratory, and integumentary systems [103,104,105,106].

The authors Guillén and Ibargoitia [26], Gonzalez Romero et al. [107], Valero et al. [25] and Villaescusa Castillo et al. [24] have published several studies on the chemical composition of *Chiliadenus glutinosus*, confirming the presence of a wide variety of phytochemicals, of which about one hundred different compounds have been described to date, mainly terpenes, esters, alkanes, lactones, flavonoids, and vitamin [24,25]. However, the main compounds are only camphor, borneol, lucinone, glutinone, kutdtriol, quercetin, and kaempferol.

### 3.1. Circulatory System

*Chiliadenus glutinosus* has been used as an infusion in localities of Jaen to treat hypotension. This blood pressure-raising effect can be associated with compounds such as camphor. The effect of this phytochemical was tested on patients with orthostatic hypotension [70]. The study suggests that the underlying hemodynamic mechanisms can be attributed to an increase in total peripheral resistance induced by an increase in the tone of the arterioles, thereby counteracting an orthostatic drop in blood pressure. This infusion has also been used with the opposite effect as a remedy for hypertension in Catalonia. It has been proven in other clinical trials that daily oral administration of quercetin in rats with induced hypertension, progressively reduces systolic pressure, which is why antagonistic effects are observed for different compounds present in the same species [79].

In other regions such as Aragon, southeast of (Castilla-La Mancha or north of Andalusia, the infusion of this plant is considered to be useful to give greater fluidity and achieve better blood circulation. It has been proven that some compounds such as borneol and quercetin have antithrombotic or anti-aggregate effects, which reduce the risk of cardiovascular diseases and thrombi. The antithrombotic effect of borneol could be due to its anticoagulant effect [78]. Flavonoids such as quercetin have also been reported to inhibit collagen-induced platelet aggregation in vitro. However, in vitro concentrations may not have the same effect as in vivo [80,108].

The *Chiliadenus glutinosus* infusion has also been used in north of Valencian Community, Region of Murcia, and southeast of Castilla-La Mancha to reduce the pain of swollen legs or improve circulation in people who suffer from varicose veins. Camphor and borneol have been shown in human clinical trials to have anti-inflammatory effects. In addition, camphor can increase blood flow locally in skin and muscle, in turn providing sensations of cold and heat, which achieves an effect of improving blood circulation and could give some relief to the aforementioned conditions [69,73]. The main mechanism proposed for the anti-inflammatory effect of borneol could be based on the fact that this compound significantly decreased the release of interleukins IL-8 and IL-6 (molecules involved in inflammatory processes [109]), with an average inhibition of 67–76% and 50–61% respectively, although other similar compounds could also have a synergistic influence. Camphor has also been shown to activate transient specific calorific potential receptors TRPV1 and TRPV3, and the human and rat cold-sensitive channel transient receptor (TRPM8). According to the prediction of the *Swiss Target Prediction* software [102] (Table 9 and Table 10), camphor can bind to carbonic anhydrase I (CA1), an enzyme responsible for the regulation of cellular pH and mediator of cerebral vascular permeability, and borneol that is known having an affinity for the TRPM8 receptor, and sensitive to temperature changes.

The infusion of this plant has also been recommended in the traditional medicine of southeast of Castilla-La Mancha) and north of Andalusia to improve the weak heart and its ailments. Trials with quercetin have been shown to have a positive effect on the recovery of myocardial contractile activity, especially after an ischemic process. This mechanism appears to be related to cytochrome P450; quercetin induces a decrease in xanthine dehydrogenase (XDH) and xanthine oxidase (XO), typical enzymes of ischemic processes [85]. The simulation in Swiss Target Prediction [102] also shows the affinity of the quercetin molecule for the XDH enzyme (Table 12), which contributes to the generation of reactive oxygen species.

Reviewing other articles, evidence of other beneficial effects on the circulatory system of some compounds present in *Chiliadenus glutinosus* has also been found. The possible antidiabetic effect of kaempferol and quercetin stands out, being able to significantly improve insulin-stimulated glucose absorption in mature adipocytes [91]. Quercetin is also mentioned as a possible preventive compound against arteriosclerosis [85] or the possible neuroprotective and vasodilator effect of kaempferol [97,101]. However, no scientific evidence has been found linking the use of *Ch. glutinosus* infusion in localities of Murcia as a treatment for anemia. According to the prediction in Swiss Target Prediction [102], quercetin and kaempferol have affinity for the enzyme NADPH oxidase 4 (NOX4), which can regulate the insulin signaling cascade (Table 12 and Table 13). Therefore, there could be a certain relationship between these compounds and their possible anti-diabetic effect, although a more exhaustive study would be necessary to know the mechanisms involved.

### 3.2. Digestive System

The infusion of *Chiliadenus glutinosus* is also widely used in much of the eastern regions of the peninsula as a remedy to treat diarrhea. This effect could be based on the spasmolytic action of quercetin [87], which blocks voltage-gated calcium channels and relaxes precontracted tissues in in vivo assays with KCl depending on the concentration. The anticonvulsant effect of borneol has also been reported in rats, causing an inhibitory effect on electrically or chemically inducible seizures [110].

Another of the best-known uses of this infusion is to treat stomach ulcers, especially in southeast of Castilla-La Mancha, Region of Murcia, and north of Andalusia. The most common cause of this type of ulcer is the bacterium *Helicobacter pylori* (Marshall et al., 1985; Goodwin et al., 1989), which can be found in the human gastrointestinal tract. The compound borneol has antimicrobial properties. It is thought that its mechanism of action is based on the disruption of the integrity of the bacterial membrane [72,111]. Other reported properties of borneol are its analgesic and anti-inflammatory effect, due to its action on the modulation of the GABA system involved in pain regulation, which could be associated with the treatment of common stomach complaints [74]. Reviewing other articles, the in vitro cytotoxic effect of camphor on human epithelial colorectal carcinoma cells has also been reported [112]. However, it has not been possible to verify or find evidence that relates the compounds present in the infusion of the plant, with the digestive, emetic, and appetite stimulant effects reported in several regions or their use to treat appendicitis in north of Aragon).

### 3.3. Genito-Urinary System

Traditionally, the infusion of *Chiliadenus glutinosus* was used to treat some conditions related to the genito-urinary system. In some towns in Region of Murcia and southeast of Castilla-La Mancha, the infusion is consumed to relieve kidney pain. This use may be related to the anti-inflammatory and analgesic effect of borneol and camphor, already discussed [69,70]. Quercetin and kaempferol could also provide anti-inflammatory effects. The infusion was reported in the Region of Murcia, as a remedy for kidney diseases (e.g., urinary infections caused by microorganisms) This use could be related to the antimicrobial activity of the compounds present in *Chiliadenus glutinosus*: camphor and borneol, together with quercetin and kaempferol [72,82,90,97,113]. Other reported uses of this infusion were as a diuretic drink in Cantabria, southeast of Castilla-La Mancha, and Region of Murcia to treat kidney stones, although no related trials or scientific evidence have been found to justify these applications. However, the Swiss Target Prediction software [102] shows as a molecular target of quercetin, the vasopressin arginine receptor (AVPR2), involved in water reabsorption in the kidneys (Table 12). So, this relationship should be studied in a way which the two components interact, and their effects on kidney function.

### 3.4. Locomotor System

In Region of Murcia and north of Andalusia the external application of *Chiliadenus glutinosus* decoction has been recommended to relieve bone pain and rheumatism. This application could be associated with the anti-inflammatory effects of compounds such as lucinone, glutinone, kutdtriol, and quercetin, or analgesic effects related to camphor and borneol [25,69,70,82]. The Swiss Target Prediction [102] software shows the glutinone affinity for the enzyme cyclooxygenase 1 (PTSG1), which enables the production of prostaglandins from arachidonic acid (Table 11). For this reason, it would be necessary to know the interaction of both elements and their effects on inflammatory processes. It can also be observed in Table 9, the affinity of camphor for carbonic anhydrase 2 (CA2), essential in the processes of bone resorption and differentiation of osteoclasts.

### 3.5. Nervous System

The infusion of *Chiliadenus glutinosus* has been used as a remedy to treat states of nervousness, elevate mood, and clear the mind in Catalonia, southeast of Castilla-La Mancha, center of Valencian Community, and Navarre. Some compounds present in *Ch. glutinosus* such as borneol, have shown sedative or anxiolytic effects that could justify the above uses. Trials were performed in mice that were induced pain with acetic acid injection and hot plate, and the results corroborated the sedative effect of borneol, due to its mentioned action on the modulation of the GABA system [74]. This infusion has also been used to treat headaches in east of Castilla-La Mancha and north of Andalusia), which could be justified by the analgesic effect of borneol, an important central and peripheral antinociceptive, and anti-inflammatory [74]. In other studies, its topical analgesic effect has been proven, relieving pain in patients with postoperative pain, since this compound induces a topical analgesia mediated by the cationic cold receiving channel TRPM8 and a descending glutamatergic mechanism in the spinal cord. Another reported effect of borneol is the neuroprotective effect. The blood–brain barrier (BBB) plays an essential role in maintaining a stable homeostatic environment, so its destruction or increased permeability are common pathological processes in many serious diseases of the central nervous system (CNS). In another study, the administration of borneol exerted a significant decrease in the permeability of the blood–brain barrier, keeping it at stable levels and having a preventive effect on possible CNS pathologies or cerebral ischemic lesions [77].

### 3.6. Respiratory System

*Chiliadenus glutinosus* infusion has also been commonly recommended throughout the eastern regions of the peninsula to treat colds and flu. This could be associated with the antimicrobial and viricidal effect of borneol [72,111]. For example, its effectiveness has been proven against herpes simplex virus 1 (HSV-1), the results showed a complete inhibition of viral replication at a concentration of 0.06%. Kaempferol is also an effective antiviral, it has been proven effective against the herpes simplex virus, cytomegalovirus, influenza virus (responsible for seasonal flu), and the human immunodeficiency virus (HIV). Kaempferol inhibits the reverse transcriptase enzyme and viral and binding proteases, stopping the viral replication of the mentioned viruses [97,114].

The infusion has also been used to treat bronchitis, asthma, or sore throat, which could be due to the anti-inflammatory and stimulating effect of camphor and borneol on the respiratory system [69,74,76].

Other trials [85,90] show that quercetin administration reduced the incidence of upper respiratory tract infections in cyclists after intensified outdoor exercise in winter and a growing literature base supports the anti-pathogenic properties of quercetin against rhinovirus, adenovirus, and coronavirus related to these respiratory infections. These studies indicate that quercetin blocks viral replication at an early stage of multiplication using several mechanisms, including inhibition of molecular coupling proteases, binding of proteins to the viral capsid, and suspension of virulence enzymes such as DNA gyrase and cell lipase [90].

### 3.7. Integumentary System

The infusion or decoction of *Chiliadenus glutinosus* has been used topically to reduce inflammation of skin pimples in Navarre. The anti-inflammatory effect may be due to the presence of lucinone, glutinone, and kutdtriol. These compounds showed anti-inflammatory activity in mouse peritoneal macrophages and inhibited cyclooxygenase 1 (PTSG1), reducing the production of prostaglandin 2 (PGE2), involved in inflammatory processes [25,74]. The Swiss Target Prediction [102] software also reveals the affinity of glutinone for PTSG1 (Table 11). The liquid resulting from the decoction of the plant has been used externally to disinfect wounds, blows or animal bites in Region of Murcia, southeast and east of Castilla-La Mancha), and north of Andalusia), which may be related and justified to the antimicrobial, antinociceptive, and anti-inflammatory properties of the borneol, and the topical sensation of cold provided by camphor, which can provide relief from these conditions [73].

### 3.8. Other Biological Activities of Interest Present in the Selected Compounds.

Reviewing the information and results of different clinical trials [25,72,77,79,84,94,115], other biological properties of the selected compounds have been found, less related to the therapeutic uses of *Chiliadenus glutinosus* in folk medicine, but of great interest for the research and development of future treatments for numerous human pathologies.

Firstly, borneol, camphor, kaempferol, and quercetin have antioxidant properties. It has been proven in vitro tests that the administration of borneol can reduce oxidative reactions and the toxicity of free radicals by increasing the activity of superoxide dismutase (SOD), a powerful antioxidant enzyme that plays a bio-protective role by alternatively catalyzing the dismutation of the superoxide radical in molecular oxygen or hydrogen peroxide in living cells [72,77]. According to other studies, kaempferol has antioxidant and protective effects against endothelial damage. Its mechanism of action may be associated with an improvement in nitric oxide production and a decrease in dimethylarginine levels [25,94]. Quercetin and its glycosylated derivatives are also capable of inhibiting lipid peroxidation in various biological systems in vitro: lipoproteins, liposomes, hepatocytes in cell cultures, and erythrocyte membranes [79,82,84,115].

Furthermore, it is worth mentioning the antiproliferative activity of quercetin. This cytotoxic action could be related to the incapacity to release lactate, the reduction of the nucleotide 5’-adenosine triphosphate (ATP), the stimulation of the production of transforming growth factor-beta 1 (TGF-b1), known for its antiproliferative activity, and blocking potassium channels. Quercetin can also prevent cancer cells from developing cellular thermotolerance, and therefore a greater efficacy could be achieved through anticarcinogenic treatment by clinical hyperthermia [80,116]. Secondly, there are studies that corroborate the antigenotoxic effect of borneol on rats’ hepatocytes and testicular cells when it is administered orally in drinking water. It was verified that borneol reversed the oxygen and glucose deprivation followed by reperfusion-induced neuronal injury, nuclear condensation, the production of reactive intracellular oxygen species (ROS), and dissipation of mitochondrial membrane potential in cortical neurons [25]. For all these reasons, it is considered of interest to deepen the study of the antioxidant and antiproliferative mechanisms of these molecules, to develop better treatments and drugs against cancer.

## 4. Material and Methods

### 4.1. Sources of Information

Firstly, an exhaustive bibliographic search was carried out to compile the medicinal uses of the *Chiliadenus glutinosus* species in Spain, the chemical composition, and previous clinical trials on the biological activity of some phytochemicals present in the species. For this, specialized databases such as Dialnet, Google Scholar, Pub Med, or ScienceDirect, were used. Keywords were used to search for information such as “traditional uses”, “ethnobotany”, “traditional medicine”, combined with the correct species name “*Chiliadenus glutinosus*”, or its synonymous plant names such as “*Jasonia glutinosa*”, “*Jasonia saxatilis* (Lam.) Guss.”, “*Erigeron glutinosus* L.”, or “*Inula saxatilis* Lam.”, and finally the names of the provinces or Autonomous Communities of Spain where the plant lives were added to these search terms. Finally, the names of the molecules present in the species such as “borneol” or “camphor”, were also used as keywords. The chemical structures of some molecules present in *Chiliadenus glutinosus* were obtained from the public domain PubChem database [117], maintained by the National Center for Biotechnology Information (NCBI) of the United States.

### 4.2. Distribution: Data Collection and Mapping

The *Chiliadenus glutinosus* distribution map was drawn up by downloading from the GBIF biodiversity data portal [28], and the references of the presence of the species in Spain. With this information, a distribution map of the species was elaborated with the ArcGIS software version 10.5.1 [27].

### 4.3. Validation Methodology

Finally, in silico modeling was performed using the Swiss Target Prediction online tool [102] to predict the targets of the bioactive molecules present in *Chiliadenus glutinosus*. This tool is based on the so-called “principle of similarity”, which establishes that two similar molecules also have similar properties. Therefore, when introducing a bioactive molecule into the tool to query the possible targets to which it can be attached, a prediction is made based on the combination of 2D and 3D similarity between a set of approximately 370,000 assets in known targets. Once the query is made, the tool displays and arranges the possible molecular targets from highest to lowest probability of binding.

## 5. Conclusions

After the corresponding review of specific literature on previous clinical trials and the in silico predictions of possible molecular targets of the phytochemicals present in higher proportions in the essential oil of *Chiliadenus glutinosus*, suggest the biological activities related to the compounds present in the plant, and outline its potential which must be confirmed by clinical studies of these majority compounds. Therefore, the potential properties provided by the presence of camphor borneol, quercetin, kaempferol lucinone, glutinone, and kutdtriol in *Ch. glutinosus*, establish a link between the therapeutic effects to a large part of the reported uses of the species to treat several conditions of the organic systems of the human being in traditional Spanish medicine.

The results place the species in a promising position to suggest future scientific research. This should focus on conducting in vitro and in vivo clinical trials testing the extracts and all the phytochemical components of the plant to gain a better understanding of their biological activity, their effects on the body, the mechanisms of action, the most appropriate form of administration and the effective doses to achieve beneficial effects on human health, which would provide a direct and much more comprehensive validation of its medicinal properties.

To conclude, this study contributes to the preservation, documentation, and validation of the medicinal uses of *Chiliadenus glutinosus* in Spain and lays the foundations for future scientific research to develop new drugs and more effective therapies to treat conditions and diseases that affect the different organ systems of the human being and opens a new way to promote the cultivation and production of the species for medicinal purposes at an industrial level.

## Figures and Tables

**Table 1:** Compilation of the medicinal uses of *Chiliadenus glutinosus* as a remedy for diseases of the circulatory system in Spain, compiled from the existing specific literature.

Remedy or Use	Form of Employment	Administration	Autonomous Community
Raise blood pressure	Infusion	Internal	Catalonia [17]
Lower blood pressure			Andalusia [17,32]
Give fluidity to the blood			Aragon [17] Castilla-La Mancha [17] Andalusia [33]
Purify the blood			Navarre [34]
Anemia			Region of Murcia [35]
Improve circulation			Region of Murcia [35] Andalusia [33]
Weak heart			Castilla-La Mancha [17]
Heart ailments			Andalusia [33]
Varicose veins			Castilla-La Mancha [17]
Relieve leg swelling	Infusion		Valencian Community [1]
	Decoction		Region of Murcia [17,35]

**Table 2:** Compilation of the medicinal uses of *Chiliadenus glutinosus* as a remedy for diseases of the digestive system in Spain, compiled from the existing specific literature.

Remedy or Use	Form of Employment	Administration	Autonomous Community
Digestive	Macerate in anise	Internal	Navarre [34]
			Catalonia [1]
	Infusion		Catalonia [36] Aragon [18,37] Navarre [38,39,40] Basque Country [22,41,42] La Rioja [43] Castile and Leon [44] Valencian Community [45,46,47,48] Castilla-La Mancha [49,50] Andalusia [33,51,52] Cantabria [53]
Diarrhea	Infusion		Aragon [54] Navarre [40] Basque Country [22,41,42] Valencian Community [1,45,46,47] Andalusia [17]
Appendicitis			Aragon [54]
Stomach ache			Navarre [34,55] Basque Country [22,41,42] Valencian Community [45,46,47] Community of Madrid [56] Castilla-La Mancha [57]
Stomach ulcers			Region of Murcia [35] Castilla-La Mancha [58] Andalusia [17,33]
Gasses			Valencian Community [1,46] Andalusia [33]
Indigestion or heaviness			Region of Murcia [35]
Stimulate the appetite			Valencian Community [47] Region of Murcia [35]
Digestive problems or indisposition (in general)			Aragon [54,59] Valencian Community [47,48] Region of Murcia [35] Balearic Islands [23] Castilla-La Mancha [49,57,58] Andalusia [21,32]
Help to vomit	Infusion (high doses)		Valencian Community [1]

**Table 3:** Compilation of the medicinal uses of *Chiliadenus glutinosus* as a remedy for diseases of the genito-urinary system in Spain, compiled from the existing specific literature.

Remedy or use	Form of Employment	Administration	Autonomous Community
Diuretic	Infusion	Internal	Region of Murcia [17] Castilla-La Mancha [17,58] Cantabria [53]
Kidney pain			Region of Murcia [17] Castilla-La Mancha [17,58]
Kidney stones			Region of Murcia [17] Castilla-La Mancha [17,58]
Kidney diseases (in general)			Region of Murcia [35]
Improve the kidney			Andalusia [33]

**Table 4:** Compilation of the medicinal uses of *Chiliadenus glutinosus* as a remedy for diseases of the locomotor system in Spain, compiled from the existing specific literature.

Remedy or Use	Form of Employment	Administration	Autonomous Community
Rheumatism/bone pain	Cocimiento	External (baths)	Andalusia [33]
	Macerated in alcohol	External (rubbing)	Region of Murcia [35]
Fatigue	Infusion	Internal	Castilla-La Mancha [57,58]

**Table 5:** Compilation of the medicinal uses of *Chiliadenus glutinosus* as a remedy for diseases of the nervous system in Spain, compiled from the existing specific literature.

Remedy or Use	Form of Employment	Administration	Autonomous Community
The nerves	Infusion	Internal	Catalonia [1] Valencian Community [45] Castilla-La Mancha [1]
Lift mood			Navarre [34,55]
Clear the mind			Navarre [34,55]
Headache			Navarre [60] Castilla-La Mancha [49] Andalusia [32]

**Table 6:** Compilation of the medicinal uses of *Chiliadenus glutinosus* as a remedy for diseases of the respiratory system in Spain, compiled from the existing specific literature.

Remedy or Use	Form of Employment	Administration	Autonomous Community
Colds and flu	Infusion/decoction	Internal	Catalonia [17,36,61] Aragon [54] Navarre [34,39] Valencian Community [45,47] Region of Murcia [17] Castilla-La Mancha [50,58] Andalusia [32,33] Cantabria [53]
Sore throats			Catalonia [17] Region of Murcia [17] Andalusia [33]
Bronchial ailments (bronchitis)	Decoction		Andalusia [33]
	Infusion		Castilla-La Mancha [17]
Asthmatic processes	Infusion		Castilla-La Mancha [17]
Asthmatic processes (bronchodilator)	Decoction vapors		Castilla-La Mancha [17,57,58]
Respiratory infections (in general)	Infusion/decoction		Balearic Islands [23]

**Table 7:** Compilation of the medicinal uses of *Chiliadenus glutinosus* as a remedy for diseases of the integumentary system in Spain, compiled from the existing specific literature.

Remedy or Use	Form of Employment	Administration	Autonomous Community
Skin pimples	Poultice (fresh leaves)	External	Navarre [34]
Disinfect wounds/bruises	Washings (infusion)		Region of Murcia [35]
	Washings (decoction)		
	Ointment		Castilla-La Mancha [17]
Insect bites	Decoction		Castilla-La Mancha [57]
Skin burns	Plaster		Castilla-La Mancha [57]
	Decoction		Castilla-La Mancha [57]
Anti-inflammatory for wounds	Infusion/Decoction		Andalusia [32]

**Table 8:** Biological activity of the compounds in higher proportions present in the essential oil of *Chiliadenus glutinosus*.

Chemical Group	Phytochemical	Biological Activity
Monoterpenes	Camphor	Anti-inflammatory [69] Antihypotensive [70,71] Antioxidant [72] Antibacterial [72] Antiparasitic [25] Topical vasodilator [73]
	Borneol	Anti-inflammatory [69,74] Antioxidant [25,72] Antibacterial [72,75] Antifungal [72,75] Antinociceptive [74] Topical analgesic [76] Neuroprotective [72] Antispasmodic [77] Choleretic [75] Tranquilizer [75] Anticoagulant [78] Antiparasitic [25]
Sesquiterpenes	Lucinone	Anti-inflammatory [25]
	Glutinone	Anti-inflammatory [25]
	Kutdtriol	Anti-inflammatory [25] Antiparasitic [25]
Flavonols	Quercetin	Antioxidant [79,80,81,82,83,84] Anti-inflammatory [82,83,85] Antiviral [85] Spasmolytic [25,86,87] Antithrombotic [88,89] Antipathogenic [85,90] Antidiabetic [91] Antidiarrheal [92] Antihypertensive [86]
	Kaempferol	Antioxidant [25,80,83,93,94] Anti-inflammatory [83,95,96] Spasmolytic [25] Antidiabetic [91] Anticancer [96,97,98,99] Cardioprotective [83,97] Osteoprotective [100] Relaxing [101]

**Table 9:** Main molecular targets of the phytochemical camphor obtained through the analysis in *Swiss Target Prediction* [102].

Target	Common Name	Uniprot ID	ChEMBL ID	Target Class	Probability
Nuclear receptor subfamily 1 group I member 3 (by homology)	NR1I3	Q14994	CHEMBL5503	Nuclear receptor	0.2
Cytochrome P450 19A1	CYP19A1	P11511	CHEMBL1978	Cytochrome P450	0.19
Carbonic anhydrase II	CA2	P00918	CHEMBL205	Lyase	0.15
Carbonic anhydrase I	CA1	P00915	CHEMBL261	Lyase	0.15
Carbonic anhydrase IV	CA4	P22748	CHEMBL3729	Lyase	0.15
Androgen Receptor	AR	P10275	CHEMBL1871	Nuclear receptor	0.10

**Table 10:** Main molecular targets of the phytochemical borneol obtained through the analysis in *Swiss Target Prediction* [102].

Target	Common Name	Uniprot ID	ChEMBL ID	Target Class	Probability
Carbonic anhydrase II	CA2	P00918	CHEMBL205	Lyase	0.36
Carbonic anhydrase I	CA1	P00915	CHEMBL261	Lyase	0.36
Carbonic anhydrase IV	CA4	P22748	CHEMBL3729	Lyase	0.36
Transient receptor potential cation channel subfamily M member 8	TRPM8	Q7Z2W7	CHEMBL1075319	Voltage-gated ion channel	0.31

**Table 11:** Main molecular targets of the phytochemical glutinone obtained through the analysis in *Swiss Target Prediction* [102].

Target	Common Name	Uniprot ID	ChEMBL ID	Target Class	Probability
Cyclooxygenase-1	PTGS1	P23219	CHEMBL221	Oxidoreductase	1
Cytochrome P450 19A1	CYP19A1	P11511	CHEMBL1978	Cytochrome P450	0.54
Cathepsin D	CTSD	P07339	CHEMBL2581	Protease	0.23

**Table 12:** Main molecular targets of the phytochemical quercetin obtained through the analysis in *Swiss Target Prediction* [102].

Target	Common Name	Uniprot ID	ChEMBL ID	Target Class	Probability
NADPH oxidase 4	NOX4	Q9NPH5	CHEMBL1250375	Enzyme	1
Vasopressin V2 receptor	AVPR2	P30518	CHEMBL1790	Family A G protein-coupled receptor	1
Aldose reductase	AKR1B1	P15121	CHEMBL1900	Enzyme	1
Xanthine dehydrogenase	XDH	P47989	CHEMBL1929	Oxidoreductase	1
Monoamine oxidase A	MAOA	P21397	CHEMBL1951	Oxidoreductase	1
Insulin-like growth factor I receptor	IGF1R	P08069	CHEMBL1957	Kinase	1

**Table 13:** Main molecular targets of the phytochemical kaempferol obtained through the analysis in *Swiss Target Prediction* [102].

Target	Common Name	Uniprot ID	ChEMBL ID	Target Class	Probability
NADPH oxidase 4	NOX4	Q9NPH5	CHEMBL1250375	Enzyme	1
Aldose reductase (by homology)	AKR1B1	P15121	CHEMBL1900	Enzyme	1
Xanthine dehydrogenase	XDH	P47989	CHEMBL1929	Oxidoreductase	1
Tyrosinase	TYR	P14679	CHEMBL1973	Oxidoreductase	1
Tyrosine-protein kinase receptor FLT3	FLT3	P36888	CHEMBL1974	Kinase	1
Carbonic anhydrase II	CA2	P00918	CHEMBL205	Lyase	1

## References

[1] Pardo de Santayana M., Morales R. (2004). Consideraciones sobre el género *Jasonia* (Compositae, Inuleae). Sistemática y usos. Acta Bot. Malacit..

[2] Gómiz García F., Morales Valverde R. (2006). Acerca de *Jasonia hesperia* Maire & Wilczek (Asteraceae) y las especies norteafricanas de este género. Acta Bot. Malacit..

[3] Guillén Bas A., Ferrer-Gallego P.P., Roselló Gimeno R., Gómez Navarro J., Laguna Lumbreras E., Peris J.B. (2013). *Jasonia glutinosa* subsp. congesta subsp. nov. (Compositae, Inuleae). Fl. Montiber..

[4] Lanfranco S., Lanfranco E., Westermeier R., Zammit M.-A., Mifsud M.-A., Xiberras J., Consell Insular de Menorca (2013). The vascular flora of the Maltese Islands. Islands and Plants: Preservation and Understanding of Flora on Mediterranean Islands.

[5] Kew Gardens, Missouri Botanical Garden The Plant List, a Working List of All Plant Species. http://www.theplantlist.org/.

[6] Species 2000; Sistema Integrado de Información Taxonómica. Catalogue of Life: 2019 Annual Checklist. http://www.catalogueoflife.org/.

[7] Muñoz Centeno L., Rico E. (2019). Chiliadenus Cass. Flora Iberica. Plantas Vasculares de la Península Ibérica e Islas Baleares. Vol. XVI (III) Compositae (Partim).

[8] De Laguna A. (1555). Pedacio Dioscorides Anazarbeo, Acerca de la Materia Medicinal y de los Venenos Mortíferos.

[9] Clusius C. (1576). Rariorum Aliquot Stirpium per Hispanias Observatarum Historia.

[10] Scalinger Institute Carolus Clusius (1526–1609): A life. https://web.archive.org/web/20050105153146/http://ub.leidenuniv.nl/Bc/scaligerinstitute/clusius/biography.html.

[11] Quer y Martínez J. (1762). Flora Española ó Historia de las Plantas que se Crían en España.

[12] Von Linné C. (1784). Parte Práctica de Botánica del Caballero Carlos Linneo.

[13] Loscos Bernal F. (1867). Tratado de Plantas de Aragón.

[14] Laguia Minguillon M.P., Hormigón Blánquez M. (1984). Los botánicos aragoneses del siglo XIX. La Ciencia y la Técnica en España Entre 1850 y 1936: Comunicaciones.

[15] Gadow H. (1897). Northern Spain.

[16] Agencia española de Medicamentos y Productos Sanitarios (2005). Real Farmacopea Española.

[17] Pardo de Santayana M., Morales R., Aceituno L., Molina M. (2014). Inventario Español de Los Conocimientos Tradicionales Relativos a La Biodiversidad.

[18] Villar L., Palacín J.M. (1994). Estudis etnobotànics al Pirineu Aragonès i les altres terres d’Osca. Semin. Estud. Univ..

[19] García-Baquero Moneo G. (2000). Especies vegetales (plantas vasculares) de interés medicinal presentes en La Rioja. Investigación Humanística y Científica en La Rioja: Homenaje a Julio Luis Fernández Sevilla y Mayela Balmaseda Aróspide.

[20] Tardío J., Pascual H., Morales R. (2005). Wild food plants traditionally used in the province of Madrid, central Spain. Econ. Bot..

[21] Benítez G., González-Tejero M.R., Molero-Mesa J. (2010). Pharmaceutical ethnobotany in the western part of Granada province (southern Spain): Ethnopharmacological synthesis. J. Ethnopharmacol..

[22] Menendez-Baceta G., Aceituno-Mata L., Molina M., Reyes-García V., Tardío J., Pardo-de Santayana M. (2014). Medicinal plants traditionally used in the northwest of the Basque Country (Biscay and Alava), Iberian Peninsula. J. Ethnopharmacol..

[23] Amengual i Vicens J.C. (2017). Flora Medicinal de les Illes Balears. Ph.D. Thesis.

[24] Villaescusa Castillo L., Diaz Lanza A.M., Faure R., Debrauwer L., Elias R., Balansard G. (1995). Two sesquiterpenoids, lucinone and glutinone, from *Jasonia glutinosa*. Phytochemistry.

[25] Valero M.S., Berzosa C., Langa E., Gómez-Rincón C., López V. (2013). *Jasonia glutinosa* D.C (“Rock Tea”): Botanical, phytochemical and pharmacological aspects. Bol. Latinoam. Caribb. Plant. Med. Aromat..

[26] Guillén M.D., Ibargoitia M.L. (1996). Volatile components obtained from the leaves of *Jasonia glutinosa*. Food Chem..

[27] Esri ArcGIS (10.5.1). https://desktop.arcgis.com/es/arcmap/10.5/get-started/setup/arcgis-desktop-quick-start-guide.htm.

[28] Global Biodiversity Information Facility (GBIF) *Chiliadenus glutinosus* Fourr. https://www.gbif.org/species/3089839.

[29] Muñoz Centeno L.M. (2003). Plantas medicinales españolas: *Jasonia glutinosa* (L.) DC. (Asteraceae) (Té de roca). Acta Bot. Malacit..

[30] Uribe-Echebarría P.M. *Jasonia glutinosa* (L.) DC. http://floragon.ipe.csic.es/ficha.php?genero=Jasonia&especie=glutinosa&subespecie=&variedad=.

[31] Aizpuru I., Aseginolaza C., Uribe-Echebarría P.M., Urrutia P., Zorrakin I. (2015). Claves Ilustradas de La Flora Del País Vasco y Territorios Limítrofes.

[32] Guzmán Tirado M.A. (1997). Aproximación a la Etnobotánica de la Provincia de Jaén. Ph.D. Thesis.

[33] Fernández Ocaña A.M. (2000). Estudio Etnobotánico en el Parque Natural de la Sierra de Cazorla, Segura y las Villas. Investigación Química de un Grupo de Especies Interesantes. Ph.D. Thesis.

[34] Akerreta S., Calvo M.I., Cavero R.Y. (2013). Sabiduría Popular y Plantas Curativas. Recopilación Extraída de un Estudio Etnobotánico en Navarra.

[35] Obón de Castro C., Rivera Nuñez D. (1991). Las Plantas Medicinales de Nuestra Región.

[36] Panareda J.M., Masnou J., Boccio M. (2010). La distribució de les plantes rupícoles al parc natural del Montseny. Monogr. Montseny.

[37] Villar Perez L. (2003). Los Saberes Científico y Popular en Torno a las Plantas del Pirineo Aragonés. Un Ejemplo de Biodiversidad Cultural.

[38] Akerreta S., Cavero R.Y., López V., Calvo M.I. (2007). Analyzing factors that influence the folk use and phytonomy of 18 medicinal plants in Navarra. J. Ethnobiol. Ethnomed..

[39] Calvo M.I., Akerreta S., Cavero R.Y. (2011). Pharmaceutical ethnobotany in the riverside of Navarra (Iberian Peninsula). J. Ethnopharmacol..

[40] Calvo M.I., Akerreta S., Cavero R.Y. (2013). The pharmacological validation of medicinal plants used for digestive problems in Navarra, Spain. Eur. J. Integr. Med..

[41] Alarcón R., Pardo-De-Santayana M., Priestley C., Morales R., Heinrich M. (2015). Medicinal and local food plants in the south of Alava (Basque Country, Spain). J. Ethnopharmacol..

[42] Menendez Baceta G. (2015). Etnobotánica de las Plantas Silvestres Comestibles y Medicinales en Cuatro Comarcas de Araba y Bizkaia. Ph.D. Thesis.

[43] Las Heras Etayo N. (2019). Estudio Etnobotánico Sobre los Usos Tradicionales de las Especies Vegetales en el Valle del Cidacos, La Rioja.

[44] Museo de Ecología Humana La Herencia del Conocimiento Tradicional de las Plantas Medicinales: Té de Roca, Sebúlcor, Segovia. http://museoecologiahumana.org/obras/la-herencia-del-conocimiento-tradicional-de-las-plantas-medicinales/.

[45] Fresquet Febrer J.L., Blanquer Roselló G., Galindo Dobón M., Gallego Estrada F., García de la Cuadra Arizo R., López Bueno J.A., Sanjosé P.A. (2001). Inventario de las plantas medicinales de uso popular en la ciudad de Valencia. Med. Cienc. Soc..

[46] Peris Gisbert J.B. (2013). Etnobotánica Farmacológica Valenciana. An. R. Acad. Med. Comunitat Valencia..

[47] Segarra Durá E. (2015). Etnobotánica farmacéutica del Campo de Turia y de los Serranos. Ph.D. Thesis.

[48] Gómez Gutiérrez A. (2014). Aproximación Etnobotánica de la Comarca Alicantina del Medio-Alto Vinalopó. Trabajo de Fin de Grado, Universitat d’Alacant, Spain. http://hdl.handle.net/10045/40164.

[49] Rojo J., García Carrero P., García López E., Pérez Badia R. (2007). Estudio Etnobotánico del Municipio de Enguídanos (Cuenca).

[50] Esteso Esteso F. (1992). Vegetación y Flora del Campo de Montiel: Interés Farmacéutico.

[51] Benítez Cruz G. (2009). Etnobotánica y Etnobiología del Poniente Granadino. Ph.D. Thesis.

[52] González-Tejero García M.R. (1989). Investigaciones Etnobotánicas en la Provincia de Granada. PhD Thesis.

[53] Pieroni A., Price L.L. (2006). Eating and Healing: Tradicional Food as Medicine.

[54] Villar Perez L., Palacin Latorre J.M., Calvo Eito C., Gomez Garcia D., Montserrat Marti G. (1987). Plantas Medicinales del Pirineo Aragonés y Demás Tierras Oscenses.

[55] Cavero R.Y., Akerreta S., Calvo M.I. (2011). Pharmaceutical ethnobotany in the middle Navarra (Iberian Peninsula). J. Ethnopharmacol..

[56] Aceituno Mata L. (2010). Estudio Etnobotánico y Agroecológico de la Sierra Norte de Madrid. Ph.D. Thesis.

[57] Verde López A. (2002). Estudio Etnofarmacológico de Tres Áreas de Montaña de Castilla-La Mancha. Ph.D. Thesis.

[58] Fajardo J., Verde A., Rivera D., Obón C. (2000). Las Plantas en la Cultura Popular de la Provincia de Albacete.

[59] Burguet Zamit J. (2017). Etnobotánica del Municipio de Alcalá de la Selva en la Sierra de Gúdar-Javalambre (Teruel). Trabajo de Fin de Grado, Universitat Politècnica de València, Spain. http://hdl.handle.net/10251/87169.

[60] Calvo M.I., Cavero R.Y. (2015). Medicinal plants used for neurological and mental disorders in Navarra and their validation from official sources. J. Ethnopharmacol..

[61] Raja D., Blanché C., Xirau J.V. (1997). Contribution to the knowledge of the pharmaceutical ethnobotany of la Segarra region (Catalonia, Iberian Peninsula*)*. J. Ethnopharmacol..

[62] PubChem Identifier: CID 2537. https://pubchem.ncbi.nlm.nih.gov/compound/2537#section=2D-Structure.

[63] PubChem Identifier: CID 64685. https://pubchem.ncbi.nlm.nih.gov/compound/64685#section=2D-Structure.

[64] PubChem Identifier: CID 5280343. https://pubchem.ncbi.nlm.nih.gov/compound/5280343#section=2D-Structure.

[65] PubChem Identifier: CID 10071029. https://pubchem.ncbi.nlm.nih.gov/compound/10071029#section=2D-Structure.

[66] PubChem Identifier: CID 44584275. https://pubchem.ncbi.nlm.nih.gov/compound/44584275#section=2D-Structure.

[67] PubChem Identifier: CID 11196115. https://pubchem.ncbi.nlm.nih.gov/compound/11196115#section=2D-Structure.

[68] PubChem Identifier: CID 5280863. https://pubchem.ncbi.nlm.nih.gov/compound/5280863#section=2D-Structure.

[69] Ehrnhöfer-Ressler M.M., Fricke K., Pignitter M., Walker J.M., Walker J., Rychlik M., Somoza V. (2013). Identification of 1,8-cineole, borneol, camphor, and thujone as anti-inflammatory compounds in a *Salvia officinalis* L. infusion using human gingival fibroblasts. J. Agric. Food Chem..

[70] Belz G.G., Loew D. (2003). Dose-response related efficacy in orthostatic hypotension of a fixed combination of D-camphor and an extract from fresh *Crataegus* berries and the contribution of the single components. Phytomedicine.

[71] Schandry R., Lindauer D., Mauz M. (2018). Blood pressure and cognitive performance after a single administration of a camphor-*Crataegus* combination in adolescents with low blood pressure. Planta Med..

[72] Su J., Chen J., Liao S., Li L., Zhu L., Chen L. (2012). Composition and biological activities of the essential oil extracted from a novel plant of *Cinnamomum camphora* Chvar. borneol. J. Med. Plants Res..

[73] Kotaka T., Kimura S., Kashiwayanagi M., Iwamoto J. (2014). Camphor induces cold and warm sensations with increases in skin and muscle blood flow in human. Biol. Pharm. Bull..

[74] Guedes da Silva Almeida J.R., Rocha Souza G., Cabral Silva J., Gomes de Lima Saraiva S.R., Gonçalves de Oliveira Júnior R., De Siqueira Quintans J.S., De Siqueira Barreto R.S., Rigoldi Bonjardim L., De Sócrates C.H.C., Quintans Junior L.J. (2013). Borneol, a bicyclic monoterpene alcohol, reduces nociceptive behavior and inflammatory response in mice. Sci. World J..

[75] Tabanca N., Kirimer N., Demirci B., Demirci F., Can Başer K.H. (2001). Composition and antimicrobial activity of the essential oils of *Micromeria cristata* subsp. phrygia and the enantiomeric distribution of borneol. J. Agric. Food Chem..

[76] Wang S., Zhang D., Hu J., Jia Q., Xu W., Su D., Song H., Xu Z., Cui J., Zhou M. (2017). A clinical and mechanistic study of topical borneol-induced analgesia. EMBO Mol. Med..

[77] Chen Z., Xu Q., Shan C., Shi Y., Wang Y., Chang R.C.-C., Zheng G. (2019). Borneol for regulating the permeability of the blood-brain barrier in experimental ischemic stroke: Preclinical evidence and possible mechanism. Oxid. Med. Cell. Longev..

[78] Li Y.H., Sun X.P., Zhang Y.Q., Wang N.S. (2008). The antithrombotic effect of borneol related to its anticoagulant property. Am. J. Chin. Med..

[79] Álvarez Castro E., Orallo Cambeiro F. (2003). Actividad biológica de los flavonoides (II). Acción cardiovascular y sanguínea. Offarm.

[80] García-Mateos R., Aguilar-Santelises L., Soto-Hernández M., Nieto-Angel R., Kite G. (2012). Total phenolic compounds, flavo-noids and antioxidant activity in the flowers of *Crataegus* spp. from México. Agrociencia.

[81] García-Mateos R., Ibarra-Estrada E., Nieto-Angel R. (2013). Antioxidant compounds in hawthorn fruits (*Crataegus* spp.) of Mexico. Rev. Mex. Biodivers..

[82] Lesjak M., Beara I., Simin N., Pintać D., Majkić T., Bekvalac K., Orčić D., Mimica-Dukić N. (2018). Antioxidant and anti-inflammatory activities of quercetin and its derivatives. J. Funct. Foods.

[83] Dabeek W.M., Ventura Marra M. (2019). Dietary quercetin and kaempferol: Bioavailability and potential cardiovascular-related bioactivity in humans. Nutrients.

[84] Ji M., Gong X., Li X., Wang C., Li M. (2020). Advanced research on the antioxidant activity and mechanism of polyphenols from *Hippophae* species. Molecules.

[85] Fariña Flores D. (2016). Obtención de Flavonoides de Plantas Superiores. Actividad Biológica. Trabajo de Fin de Grado, Universidad de La Laguna, Santa Cruz de Tenerife, Spain. http://riull.ull.es/xmlui/handle/915/2674.

[86] Morales M.A., Lozoya X. (1994). Calcium-antagonists effects of quercetin on aortic smooth muscle. Planta Med..

[87] Ventura-Martínez R., Ángeles-López G.E., Rodríguez R., González-Trujano M.E., Déciga-Campos M. (2018). Spasmolytic effect of aqueous extract of *Tagetes erecta* L. flowers is mediated through calcium channel blockade on the guinea-pig ileum. Biomed. Pharmacother..

[88] Janssen P.K., Mensink R.P., Cox F.J.J., Harryvan J.L., Hovenier R., Hollman P.C., Katan M.B. (1998). Effects of the flavo-noids quercetin and apigenin on hemostasis in healthy volunteers: Results from an in vitro and a dietary supplement study. Am. J. Clin. Nutr..

[89] Hubbard G.P., Wolffram S., Lovegrove J.A., Gibbins J.M. (2004). Ingestion of quercetin inhibits platelet aggregation and essential components of the collagen-stimulated platelet activation pathway in humans. J. Thromb. Haemost..

[90] Nieman D.C., Henson D.A., Gross S.J., Jenkins D.P., Davis J.M., Murphy E.A., Carmichael M.D., Dumke C.L., Utter A.C., Mcanulty S.R. (2007). Quercetin reduces illness but not immune perturbations after intensive exercise. Med. Sci. Sports Exerc..

[91] Fang X.K., Gao J., Zhu D.N. (2008). Kaempferol and quercetin isolated from *Euonymus alatus* improve glucose uptake of 3T3-L1 cells without adipogenesis activity. Life Sci..

[92] Zhang W., Chen B., Wang C., Zhu Q., Mo Z. (2003). Mechanism of quercetin as an antidiarrheal agent. Di Yi Jun Yi Da Xue Xue Bao.

[93] Jun S.P., Ho S.R., Duck H.K., Ih S.C. (2006). Enzymatic preparation of kaempferol from green tea seed and its antioxidant activity. J. Agric. Food Chem..

[94] Singh R., Singh B., Singh S., Kumar N., Kumar S., Arora S. (2008). Anti-free radical activities of kaempferol isolated from *Acacia nilotica* (L.) Willd. Toxicol. Vitr..

[95] Devi K.P., Malar D.S., Nabavi S.F., Sureda A., Xiao J., Nabavi S.M., Daglia M. (2015). Kaempferol and inflammation: From chemistry to medicine. Pharmacol. Res..

[96] Imran M., Rauf A., Shah Z.A., Saeed F., Imran A., Arshad M.U., Ahmad B., Bawazeer S., Atif M., Peters D.G. (2018). Chemo-preventive and therapeutic effect of the dietary flavonoid kaempferol: A comprehensive review. Phyther. Res..

[97] Calderón-Montaño J.M., Burgos-Morón E., Pérez-Guerrero C., López-Lázaro M. (2011). A Review on the dietary flavonoid kaempferol. Mini Rev. Med. Chem..

[98] Chen A.Y., Chen Y.C. (2013). A Review of the dietary flavonoid, kaempferol on human health and cancer chemoprevention. Food Chem..

[99] Imran M., Salehi B., Sharifi-rad J., Gondal T.A., Arshad M.U., Khan H., Guerreiro S.G. (2019). Kaempferol: A key emphasis to its anticancer potential. Molecules.

[100] Wong S.K., Chin K.Y., Ima-Nirwana S. (2019). The osteoprotective effects of kaempferol: The evidence from in vivo and in vitro studies. Drug Des. Dev. Ther..

[101] Gorginzadeh M., Vahdat M. (2015). Smooth muscle relaxant activity of *Crocus sativus* (saffron) and its constituents: Possible mechanisms. Avicenna J. Phytomed..

[102] Swiss Institute of Bioinformatics (SIB) Swiss Target Prediction. http://swisstargetprediction.ch/.

[103] Aguilera Carbonell L.M., Casas Úbeda J.M., Cerezo Gallego J.M., Chaves González F., Garrido Garrido J.L., Javaloyes Tarí E., Majadas García A., Martín Peña A., Romero Sánchez J., Vives Boix F. (2005). La Enciclopedia Del Estudiante—09 Ciencias de La Vida.

[104] Sepúlveda Saavedra J. (2015). Texto Atlas Histología Biología Celular y Tisular.

[105] Junta de Castilla y León—Consejería de Salud Nuestro Aparato Respiratorio: ¿Cómo es y Cómo Funciona?. https://www.saludcastillayleon.es/AulaPacientes/es/guia-asma/aparato-respiratorio-funciona.

[106] Página web Concepto Definición Sistema Genitourinario. https://conceptodefinicion.de/sistema-genitourinario/.

[107] González Romero M.A., Villaescusa Castillo L., Díaz Lanza A.M., Arribas Bricio J.M., Soria Monzón C.A., Sanz Perucha J. (2003). Volatile composition of *Jasonia glutinosa* D. C. Z. Nat..

[108] Knekt P., Isotupa S., Rissanen H., Heliövaara M., Järvinen R., Häkkinen S., Aromaa A., Reunanen A. (2000). Quercetin intake and the incidence of cerebrovascular disease. Eur. J. Clin. Nutr..

[109] Mastinu A., Bonini S.A., Premoli M., Maccarinelli G., Mac Sweeney E., Zhang L., Lucini L., Memo M. (2021). Protective effects of *Gynostemma pentaphyllum* (var. Ginpent) against lipopolysaccharide-induced inflammation and motor alteration in mice. Molecules.

[110] Álvarez Leyva C.A. (1992). Valoración de la Actividad Anticonvulsionante del Borneol en Rata Wistar. Tesis Profesional.

[111] Eryiğit T., Okut N., Ekici K., Yildirim B. (2014). Chemical composition and antibacterial activities of *Juniperus horizontalis* essential oil. Can. J. Plant Sci..

[112] Petrović A., Milutinović M.M., Petri E.T., Živanović M., Milivojević N., Puchta R., Scheurer A., Korzekwa J., Klisurić O.R., Bogojeski J. (2019). Synthesis of camphor-derived bis(pyrazolylpyridine) rhodium(III) complexes: Structure-reactivity relation-ships and biological activity. Inorg. Chem..

[113] Lax Vivancos V. (2014). Estudio de la Variabilidad Química, Propiedades Antioxidantes y Biocidas de Poblaciones Espontáneas de *Rosmarinus officinalis* L. en la Región de Murcia. Ph.D. Thesis.

[114] Armaka M., Papanikolaou E., Sivropoulou A., Arsenakis M. (1999). Antiviral properties of isoborneol, a potent inhibitor of herpes simplex virus type 1. Antivir. Res..

[115] Sánchez Gallego J.I. (2009). Efecto de la Quercetina y la Rutina Frente al Daño Oxidativo Inducido en Eritrocitos con Distintos Con-Tenidos de Colesterol. Ph.D. Thesis.

[116] Álvarez Castro E., Orallo Cambeiro F. (2003). Actividad biológica de los flavonoides (I). Acción frente al cáncer. Offarm.

[117] PubChem. https://pubchem.ncbi.nlm.nih.gov/.

